# The ARTIST Post‐Market Study: Evaluating a High G' Hyaluronic Acid (HA) Injectable for Chin Combined With Midface HA Filler Treatments

**DOI:** 10.1111/jocd.70807

**Published:** 2026-04-01

**Authors:** Andreas Nikolis, Jason K. Rivers, Nathan Rosen, Felipe Weinberg, Inna Prygova, Torun Bromée

**Affiliations:** ^1^ Victoria Park Medispas Montreal Quebec Canada; ^2^ Division of Plastic Surgery McGill University Montreal Quebec Canada; ^3^ Pacific Derm Vancouver British Columbia Canada; ^4^ Department of Dermatology and Skin Science University of British Columbia Vancouver British Columbia Canada; ^5^ Dermetics Cosmetic Dermatology Burlington Ontario Canada; ^6^ Galderma Dallas Texas USA; ^7^ Galderma Uppsala Sweden

**Keywords:** chin, hyaluronic acid filler, lower face, midface, *Restylane Shaype*

## Abstract

**Background:**

A novel hyaluronic acid (HA) injectable (Restylane Shaype, HA_SHA_) with high strength/firmness and enhanced HA concentration, optimized for lower face shaping has been developed.

**Aims:**

This prospective, 8‐week, multicenter, post‐market study evaluated three treatment algorithms, i.e., the Shayping Technique, for subjects with multiple aesthetic needs, including HA_SHA_ for chin augmentation alone/in combination with treatments for the lower face and midface with HA_DEF_ (Restylane Defyne) and HA_LYF_ (Restylane Lyft Lidocaine).

**Patients/Methods:**

Subjects ≥ 18 years were assigned to one of the following treatments: Group 1: HA_SHA_ (chin); Group 2: HA_SHA_ (chin) and HA_DEF_ (marionette line areas laterally to the chin); Group 3: HA_SHA_ (chin), HA_DEF_ (marionette line areas laterally to the chin), and HA_LYF_ (midface and/or nasolabial fold [NLF, piriform fossa] and/or jawline). Assessments included Global Aesthetic Improvement Scale (GAIS), subject and investigator satisfaction, and collection of adverse events (AEs).

**Results:**

The GAIS responder rate (“improved,” “much improved,” or “very much improved”) was 100% in all groups at Week 8 (primary endpoint) and Week 4 as assessed by the investigator and by subject self‐assessment at Weeks 4 and 8. Overall subject and investigator satisfaction with treatment was high, and the safety profile for all product combinations was favorable, with no unexpected AEs.

**Conclusions:**

Treatment with HA_SHA_ for chin augmentation alone/in combination with HA_DEF_ and HA_LYF_ for comprehensive lower face and midface lift was safe and effective, with 100% aesthetic improvement at 8 weeks post‐injection. The Shayping Technique provides standardized guidance for practitioners to perform safe injections and achieve optimal aesthetic outcomes.

## 1 Introduction

While the clinical performance of hyaluronic acid (HA) fillers is well established, there is a lack of standardized clinical guidance for safe and optimized treatment approaches to meet the needs of the real‐world aesthetic patient. In clinical practice, such patients often require treatments across multiple anatomical locations using a combination of several products. This study, the ARTIST study (A Restylane Treatment algorithm approach for subjects with appearance of Insufficient bone Structure), aimed to evaluate three treatment algorithms, collectively referred to as the Shayping Technique, targeting subjects with various aesthetic needs including chin augmentation. Specifically, the study objective was to assess the aesthetic improvement after treating the chin region with HA_SHA_ alone or in combination with HA_DEF_ for soft tissue deficiencies lateral to the chin (marionette lines) and HA_LYF_ for a midface lift (including the piriform fossa) and/or jawline, according to one of the three algorithms. In addition, the Shayping Technique offers standardized guidance on safe injections to support practitioners to achieve optimal outcomes in patients with diverse needs in clinical practice.

In Canada, a new HA injectable, HA_SHA_ (Restylane Shaype, Galderma, Uppsala, Sweden), was recently approved for temporary chin augmentation [1]. It is characterized by high strength/firmness, with a G prime of 916 Pa (0.1 Hz) and an HA concentration of 25 mg/mL [2]. Its high G prime allows for a higher degree of projection with lower injection volumes, making it optimal for placement on the periosteum [3]. It is produced using the NASHA‐HD technology, which employs efficient cross‐linking while maintaining a low degree of modification [2]. HA_SHA_ has previously been demonstrated to be safe and effective in a pivotal clinical investigation [2].

The two other HA filler products used in this study, HA_DEF_ (Restylane Defyne, Galderma, Uppsala, Sweden) [4] and HA_LYF_ (Restylane Lyft Lidocaine, Galderma, Uppsala, Sweden) [5], are also approved for market use in Canada and have been demonstrated to be safe and effective for aesthetic use in several pivotal clinical investigations [6, 7, 8, 9, 10, 11].

## 2 Materials and Methods

### 2.1 Study Design

This 8‐week prospective, post‐market, open‐label study was conducted at 3 investigational centers in Canada (NCT06428214) and utilized a 3‐arm parallel group design. Subjects enrolled were male or non‐pregnant, non‐breastfeeding females aged ≥ 18 years, seeking treatment for temporary augmentation in the chin region, with or without the need for improvement of surrounding soft face deficiencies and midface lift. Subjects had to abstain from other facial, plastic surgical, cosmetic procedures, or implant for the duration of the study. Approximately 45 subjects were to be enrolled equally into 3 treatment groups, aiming for 10 to 20% male subjects.

Exclusion criteria included a history of facial surgery below the level of the orbital rim, or prior permanent or semi‐permanent facial tissue augmentation in this facial area. Subjects were also excluded if they had received HA filler or collagen filler within 12 months or botulinum toxin or certain aesthetic procedures within 6 months in this facial area.

Study conduct complied with the international standard for clinical studies of medical devices involving human subjects, Canadian regulations and the International Council for Harmonisation of Technical Requirements for Pharmaceuticals for Human Use Good Clinical Practice. Approval was obtained from the appropriate Institutional Review Boards, and all subjects provided informed consent.

### 2.2 Treatment

**FIGURE 1:**
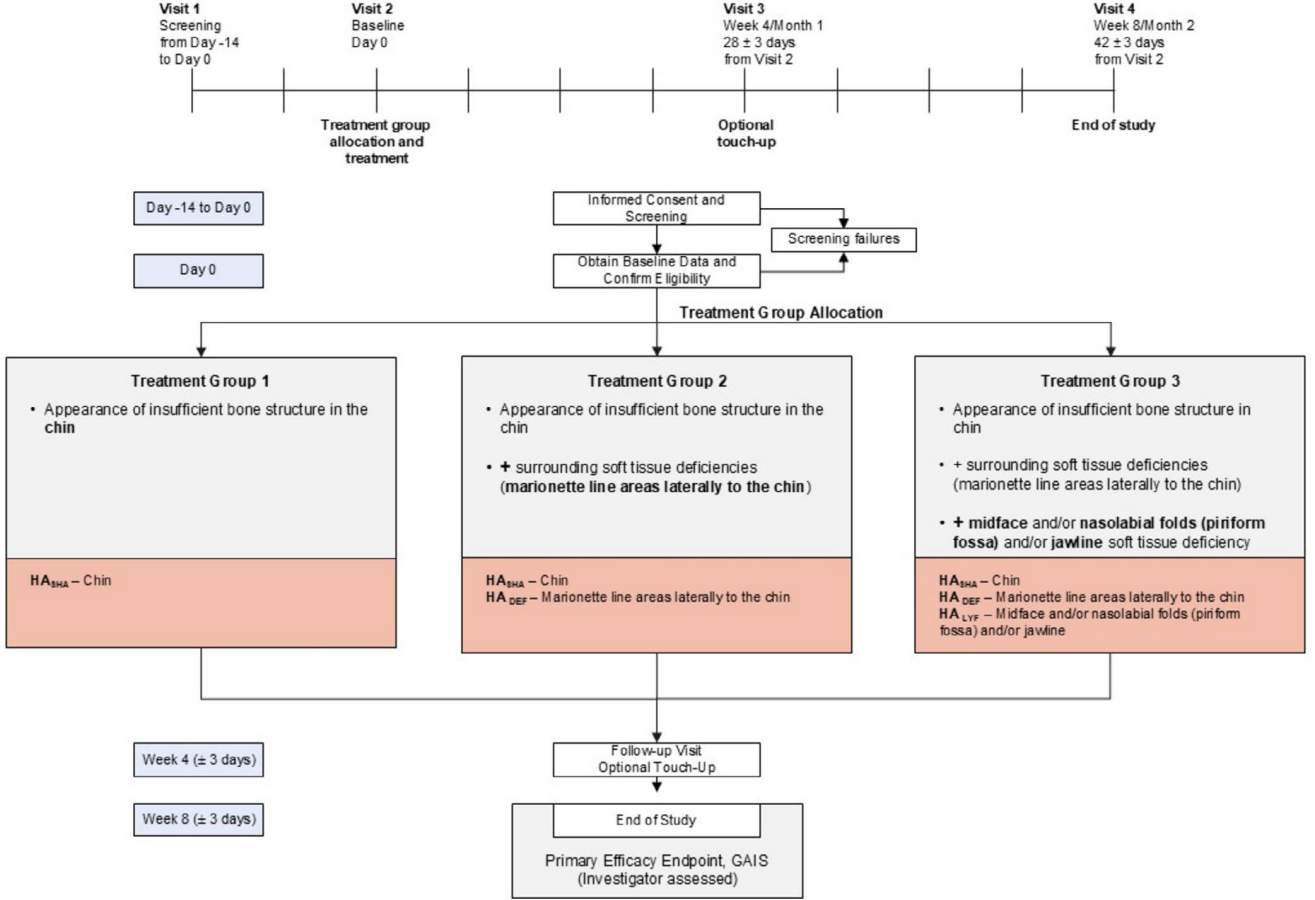
Clinical study flow chart. GAIS, Global aesthetic improvement scale.

Subjects were assigned to one of three treatment groups according to their aesthetic needs (Figure 1), and treated according to the treatment algorithm assigned to that group (Table 1):

- Group 1—Appearance of insufficient bone structure in the chin: treatment with HA_SHA_ (chin);
- Group 2—Appearance of insufficient bone structure in the chin + surrounding soft tissue deficiencies (marionette line areas laterally to the chin): treatment with HA_SHA_ (chin) and HA_DEF_ (marionette line areas laterally to the chin);
- Group 3—Appearance of insufficient bone structure in the chin + surrounding soft tissue deficiencies (marionette line areas laterally to the chin) + midface and/or nasolabial fold (NLF; piriform fossa) and/or jawline soft tissue deficiency: treatment with HA_SHA_ (chin), HA_DEF_ (marionette line areas laterally to the chin), and HA_LYF_ (midface and/or NLF [piriform fossa] and/or jawline).

**TABLE 1:** Recommended treatment plan according to the Shayping Technique.

Treatment Group 1: HA_SHA_ (chin)	Chin retrusion correction (projection, elongation, and augmentation), only at the supraperiosteal plane, according to the IFU. Needle only. Injected volume of HA_SHA_ should not exceed 4 mL at baseline treatment and not exceed 6 mL for baseline and touch‐up treatment together.
Treatment Group 2: HA_SHA_ (chin) and HA_DEF_ (marionette line areas laterally to the chin)	First, chin retrusion correction (projection, elongation, and augmentation), only at the supraperiosteal plane. For HA_SHA_ injection instructions, see Treatment group 1. Second, transition in the marionette line and prejowl region lateral to the chin at the subcutaneous plane (needle or cannula), according to the IFU. A total maximum volume of 1 mL of HA_DEF_ is recommended for marionette lines per treatment session and 2 mL for baseline and touch‐up combined.
Treatment Group 3: HA_SHA_ (chin), HA_DEF_ (marionette line areas laterally to the chin), and HA_LYF_ (midface and/or nasolabial fold [NLF, piriform fossa] and/or jawline).	First, midface restoration of structural support of the midface from lateral to medial (zygomatic arc to pyriform region), needle only at supraperiosteal plane. HA_LYF_ administered as per the IFU. The maximum volume of HA_LYF_ to be administered at baseline is 2 mL per treatment site (midface [including NLF, only in the piriform fossa] and jawline) and at the touch‐up 2 mL per treatment site, giving a maximum total of 16 mL of HA_LYF_ to be injected for baseline and touch‐up combined. Second, chin retrusion correction (projection, elongation, and augmentation), only at the supraperiosteal plane. For HA_SHA_ injection instructions, see Treatment group 1. Third, jawline definition with HA_LYF_ (recommended with cannula) at subcutaneous plane, correction of the mandibular angle (recommended with needle) at a supraperiosteal plane. Fourth, transition in the marionette line and prejowl region lateral to the chin at the subcutaneous plane (needle or cannula), see injection instructions for HA_DEF_ for Group 2.

Study products were injected at baseline according to the Instructions for Use for each product, with an optional touch‐up at Week 4 for optimal aesthetic improvement. Table 1 summarizes the recommended treatment plan for each group, including the order of filler injections and maximum recommended volumes.

### 2.3 Study Endpoints and Assessments

The primary objective was to evaluate aesthetic improvement of treated areas for each group, based on investigator‐assessed responder rate on the 7‐graded Global Aesthetic Improvement Scale (GAIS) at Week 8 (primary endpoint). A responder was defined as a subject assessed as at least improved (“improved,” “much improved,” or “very much improved”). Other response options on the GAIS were: “no change”, “worse”, “much worse”, “very much worse”.

Secondary objectives were to evaluate aesthetic improvement on the GAIS by the investigator at Week 4 and by subjects at Weeks 4 and 8, and satisfaction with study treatment at Week 8 using a subject satisfaction questionnaire (SSQ) and an investigator satisfaction questionnaire (ISQ). Response options on the satisfaction questionnaires included: “strongly disagree”, “disagree”, “neither agree or disagree”, “agree”, “strongly agree”; “very satisfied”, “satisfied”, “neither satisfied or dissatisfied”, “dissatisfied”, “very dissatisfied”; or “Yes”, “No”.

Safety was evaluated using standard collection of adverse events (AEs) throughout the study.

Exploratory evaluation of HA_SHA_ implant distribution, projection, and other characteristics was performed by using ultrasonography at baseline (post‐treatment), Week 4 (post‐treatment), and Week 8, and 3D camera at baseline (pre‐ and post‐treatment), Week 4 (pre‐ and post‐treatment), and Week 8.

### 2.4 Statistical Analyses

Data were analyzed descriptively, with effectiveness results presented using the full analysis set (FAS), i.e., all subjects who completed at least one treatment session; and safety results presented for all subjects injected with study product at least once. Sample size was not formally calculated but was based on previous experience from similar studies.

## 3 Results

### 3.1 Subjects and Treatments

Of 47 subjects screened, 45 subjects (15 per treatment group) were enrolled, treated, and completed the study. All 45 treated subjects were analyzed for effectiveness and safety.

Table 2 summarizes the demographics and baseline characteristics per group. The subject population had a mean age of 42.8 years (range: 25–74 years) and was mainly women (84%).

**TABLE 2:** Demographics.

	Group 1 (*N* = 15)	Group 2 (*N* = 15)	Group 3 (*N* = 15)	Total (*N* = 45)
Age at baseline (years)				
Mean (min–max)	36.5 (25–51)	45.9 (28–69)	46.1 (26–74)	42.8 (25–74)
Sex at birth, *n* (%)				
Female	13 (86.7)	14 (93.3)	11 (73.3)	38 (84.4)
Male	2 (13.3)	1 (6.7)	4 (26.7)	7 (15.6)
Ethnicity, *n* (%)				
Not Hispanic or Latino	15 (100.0)	14 (93.3)	15 (100.0)	44 (97.8)
Hispanic or Latino	0	1 (6.7)	0	1 (2.2)
Race, *n* (%)				
White	13 (86.7)	10 (66.7)	12 (80.0)	35 (77.8)
Asian	2 (13.3)	3 (20.0)	1 (6.7)	6 (13.3)
Other	0	2 (13.3)	1 (6.7)	3 (6.7)
Multiple^a^	0	0	1 (6.7)	1 (2.2)
Fitzpatrick skin type, *n* (%)				
I	1 (6.7)	0	0	1 (2.2)
II	3 (20.0)	3 (20.0)	5 (33.3)	11 (24.4)
III	10 (66.7)	7 (46.7)	3 (20.0)	20 (44.4)
IV	1 (6.7)	3 (20.0)	7 (46.7)	11 (24.4)
V	0	2 (13.3)	0	2 (4.4)

*Note:* Full Analysis Set.

Abbreviations: *n*, number of subjects in specific category; *N*, number of subjects in full analysis set.

^a^ White, Black or African American.

A summary of the HA filler treatments in each group, including injected volumes of each product at each treatment session, is given in Table 3. Details about injection depth and injection methods are provided in Table 4.

**TABLE 3:** HA product mean volumes injected per subject.

			Baseline	Touch‐up	Total (Baseline + touch‐up)
HA_SHA_					
	Group 1 (*N* = 15)	Mean (SD), mL	1.61 (1.037)	0.88 (0.533)	2.14 (1.267)
		*n*	15	9	15
	Group 2 (*N* = 15)	Mean (SD), mL	1.25 (0.818)	0.88 (0.493)	1.72 (0.874)
		*n*	15	8	15
	Group 3 (*N* = 15)	Mean (SD), mL	1.34 (0.881)	0.95 (0.386)	1.97 (1.193)
		*n*	15	10	15
HA_DEF_					
	Group 2 (*N* = 15)	Mean (SD), mL	0.91 (0.154)	0.76 (0.328)	1.37 (0.532)
		*n*	15	9	15
	Group 3 (*N* = 15)	Mean (SD), mL	0.87 (0.158)	0.83 (0.275)	1.37 (0.547)
		*n*	15	9	15
HA_LYF_					
	Group 3 (*N* = 15)	Mean (SD), mL	3.11 (1.333)	2.46 (1.737)	4.75 (2.959)
		*n*	15	10	15

*Note:* Safety Analysis Set.

Abbreviations: *n*, number of subjects treated; *N*, number of subjects in treatment group; SD, standard deviation.

**TABLE 4:** Overall treatment administration characteristics for each HA product.

			*n* (%)
HA_SHA_	Number of subjects injected		45
	Needle size	27‐gauge × ¾ inch (19 mm) ultra‐thin wall, TSK	45 (100)
		Other	5 (11.11)
	Injection method(s) used^a^	Bolus	31 (68.89)
		Bolus, linear threading	1 (2.22)
		Bolus, serial puncture	3 (6.67)
		Serial puncture	16 (35.56)
	Depth of injection^a^	Supraperiosteal	45 (100)
HA_DEF_	Number of subjects injected		30
	Needle/cannula size	27‐gauge blunt cannula	4 (13.33)
		27‐gauge × ¾ inch (19 mm) Ultra‐Thin Wall, TSK	8 (26.67)
		Other	22 (73.33)
	Injection method(s) used^a^	Antegrade linear threading	1 (3.33)
		Antegrade linear threading, fanning	1 (3.33)
		Antegrade linear threading, fanning, retrograde linear threading	3 (10.00)
		Fanning	9 (30.00)
		Fanning, retrograde linear threading	7 (23.33)
		Retrograde linear threading	16 (53.33)
		Retrograde linear threading, serial puncture	6 (20.0)
		Serial puncture	13 (43.33)
	Depth of injection^a^	Subcutaneous	30 (100)
HA_LYF_	Number of subjects injected		15
	Needle/cannula size	25‐gauge blunt cannula	9 (60.00)
		27‐gauge × ½ inch thin wall	15 (100)
		Other	8 (53.33)
	Injection method(s) used^a^	Bolus	11 (73.33)
		Bolus, other	6 (40.00)
		Fanning	1 (6.67)
		Fanning, retrograde linear threading	1 (6.67)
		Retrograde linear threading	9 (60.00)
		Retrograde linear threading, serial puncture	1 (6.67)
		Serial puncture	1 (6.67)
		Serial puncture, bolus	2 (13.33)
		Serial puncture, bolus, other	1 (6.67)
		Other	5 (33.33)
	Depth of injection^a^	Subcutaneous	10 (66.67)
		Supraperiosteal	15 (100)
		Other	3 (20.00)

*Note:* Safety Analysis Set.

^a^ Injector was to check all that applied.

### 3.2 Primary Outcome

#### 3.2.1 Investigator‐Reported Aesthetic Improvement at Week 8

**FIGURE 2:**
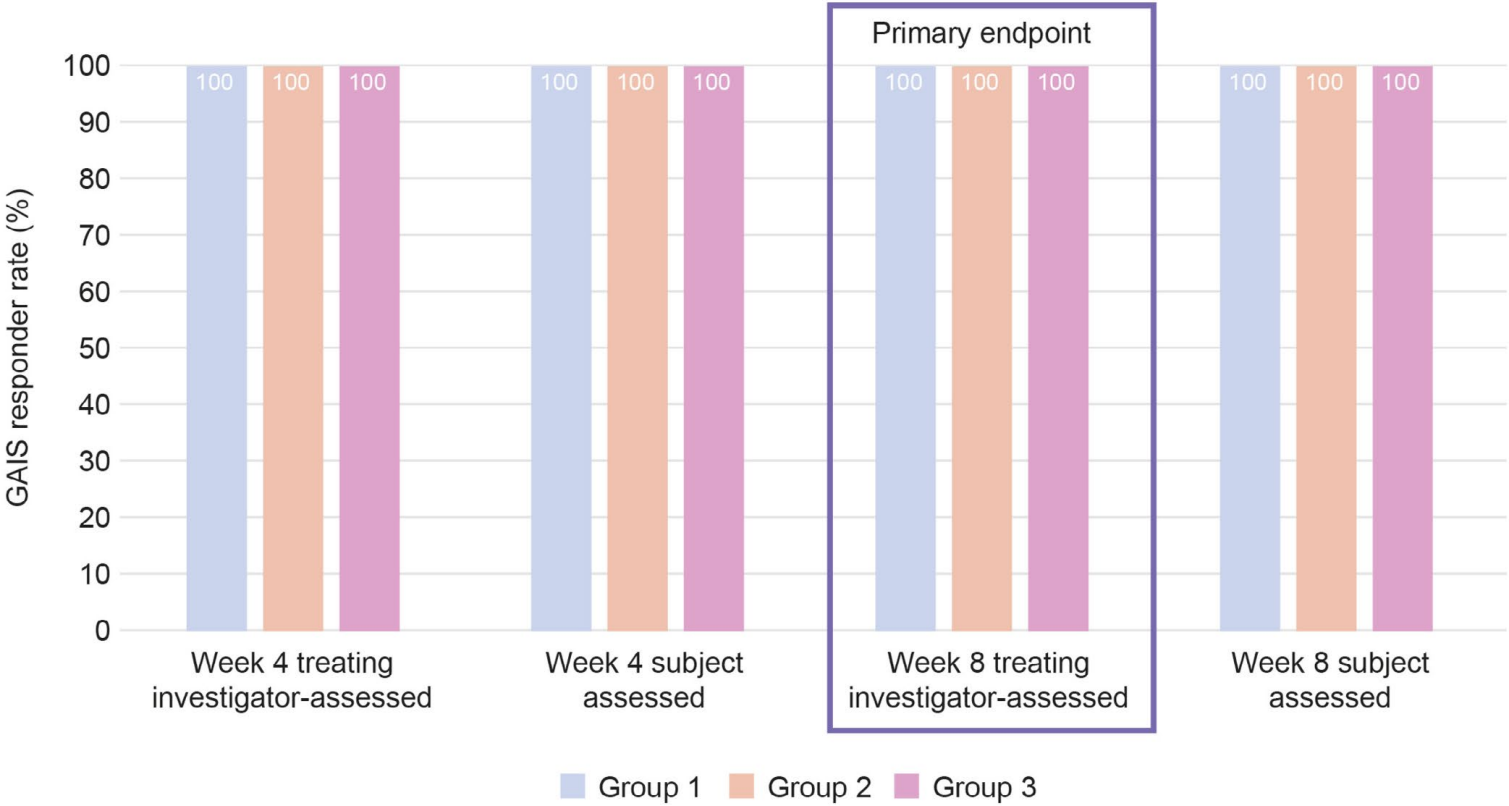
GAIS responder rates in each group at Weeks 4 and 8, according to subject assessment and treating investigator assessment (Full analysis set). Confidence interval (CI) calculated using Clopper‐Pearson method (based on binomial distribution). 95% CI: 78.2, 100 for all. GAIS, Global aesthetic improvement scale; A responder was defined as a subject graded as “improved”, “much improved”, or “very much improved” on the GAIS.

Treating investigators reported aesthetic improvement in all subjects in all 3 treatment groups at Week 8, based on the GAIS responder rates (100%; 95% CI: 78.2, 100) (Figure 2).

### 3.3 Secondary Outcomes

#### 3.3.1 Investigator‐Reported Aesthetic Improvement at Week 4 and Subject‐Reported Aesthetic Improvement at Weeks 4 and 8

All subjects also had aesthetic improvement at Week 4 as reported by investigators and at Weeks 4 and 8 as reported by the subjects themselves, with GAIS responder rates of 100% (95% CI: 78.2, 100) at each time point in all groups (Figure 2).

#### 3.3.2 Subject Satisfaction Questionnaire (SSQ)

**FIGURE 3:**
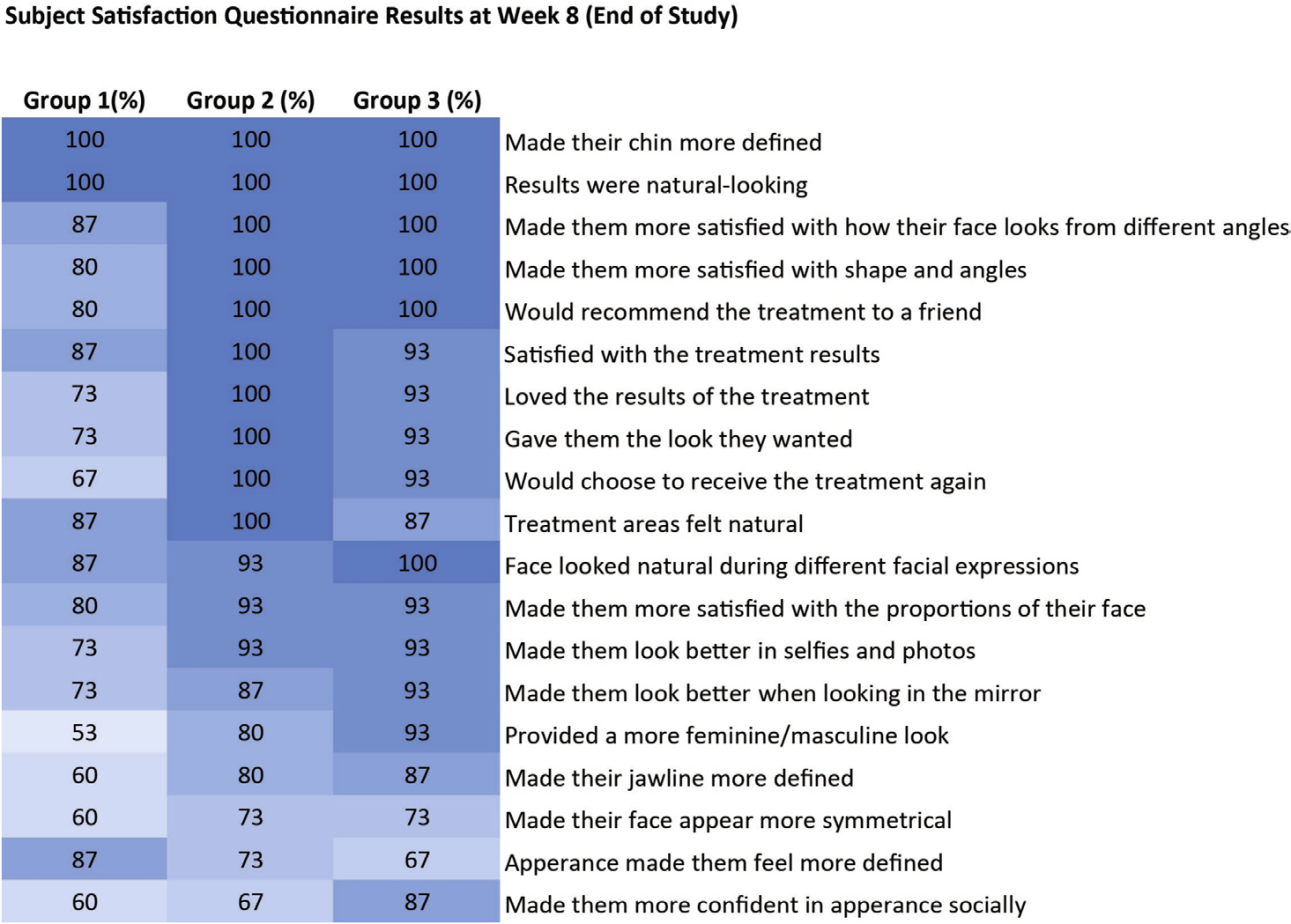
Subject Satisfaction Questionnaire (Full Analysis Set).

In all 3 treatment groups, subjects reported an overall high satisfaction with treatment results (≥ 87%) at Week 8 (Figure 3), and all subjects (100%) in all groups agreed that the treatment results looked natural and made their chin more defined.

#### 3.3.3 Investigator Satisfaction Questionnaire (ISQ)

**FIGURE 4:**
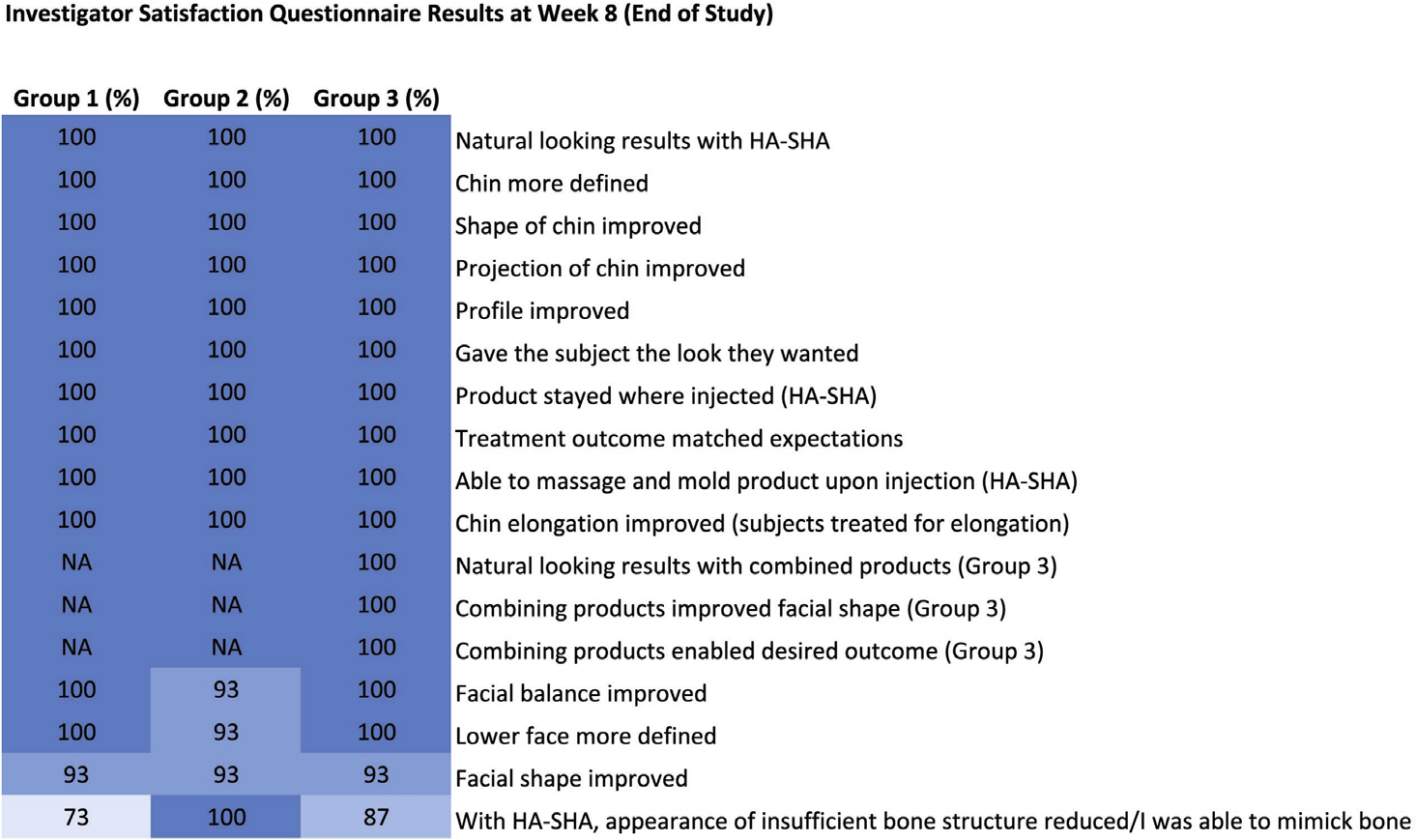
Investigator Satisfaction Questionnaire (Full Analysis Set). HA‐SHA, Restylane Shaype.

Investigator satisfaction was high at Week 8, with all subjects (100%) in all groups assessed to have natural looking results, an improved profile, and improved chin definition, shape, and projection (Figure 4). In addition, investigators reported for all subjects (100%) that HA_SHA_ stayed where injected, could be massaged and molded immediately after injection to achieve the desired results, and that the treatment outcome matched their expectations. The treatment with HA_SHA_ was able to reduce the appearance of insufficient bone structure, i.e., treatment mimicked bone in 73.3%–100% of subjects in the different groups, and 93%–100% of subjects in each group showed improvements in facial balance and lower face definition. The combination treatment with HA_SHA_, HA_DEF_ and HA_LYF_ improved the facial shape and achieved the desired outcome, with natural‐looking results in all subjects in Group 3.

#### 3.3.4 Ultrasonography and 3D Imaging

**FIGURE 5:**
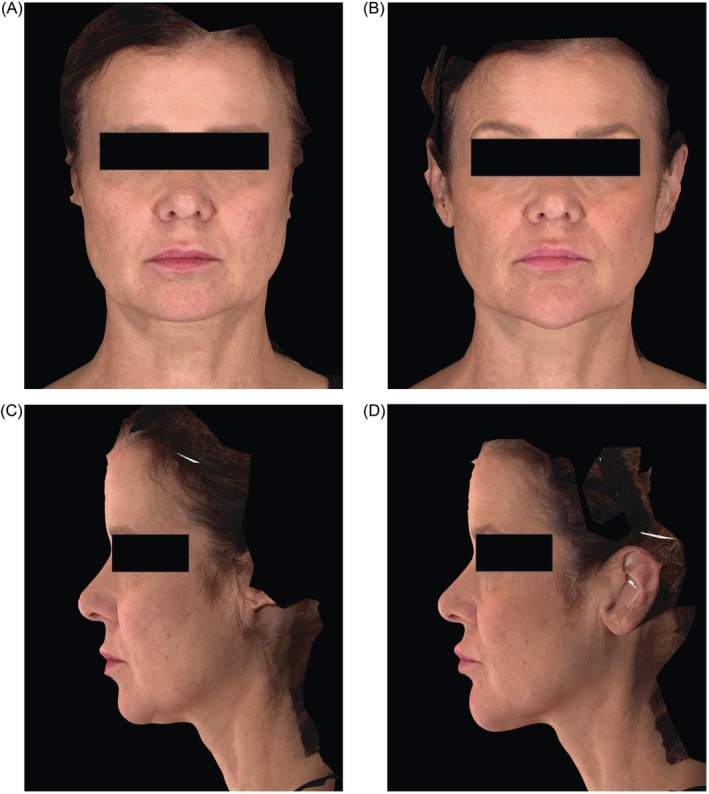
Photographs of a 53‐year‐old female subject from Group 2 (receiving HA_SHA_ and HA_DEF_ treatment); (A, C) at baseline before treatment and (B, D) at Week 8.

Example photographs of a subject before and after treatment with HA_SHA_ are shown in Figure 5. Ultrasound images (not shown) indicated that HA_SHA_ integrates into the chin tissue over time, transitioning from large to smaller aggregates while maintaining projection to the chin.

#### 3.3.5 Treatment‐Related Adverse Events

AEs with a possible relationship to treatment (products or injection procedures) occurred in 15/45 subjects (33%; 33 events) (Table 5). Except for 1 severe implant site pain in Group 1, all other treatment‐related AEs were mild or moderate. The most common types of treatment‐related AEs were implant site pain and implant site swelling.

**TABLE 5:** Treatment‐related AEs.

	Group 1 HA_SHA_ (*N* = 15)		Group 2 HA_SHA_ + HA_DEF_ (*N* = 15)		Group 3 HA_SHA_ + HA_DEF_ + HA_LYF_ (*N* = 15)	
	*n* (%)	Events	*n* (%)	Events	*n* (%)	Events
Subjects with any related AE	5 (33.3)	15	4 (26.7)	8	6 (40.0)	10
Implant site pain	5 (33.3)	6	3 (20.0)	3	4 (26.7)	5
Implant site swelling	4 (26.7)	4	2 (13.3)	2	3 (20.0)	3
Implant site bruising	0	0	3 (20.0)	3	1 (6.7)	1
Presyncope	3 (20.0)	4	0	0	0	0
Implant site erythema	1 (6.7)	1	0	0	0	0
Headache	0	0	0	0	1 (6.7)	1

*Note:* For each preferred term, total subjects were counted only once. Treatment‐related, a reasonable possibility to be caused by the study product or injection procedure.

Abbreviations: AE, adverse event; *n*, number of subjects in specific category; *N*, number of subjects in the safety analysis set.

No implant site masses or nodules were reported. Higher rates of implant site pain and implant site swelling were observed for subjects who received an injection volume of HA_SHA_ > 1.8 mL (36.4% and 27.3%, respectively) compared with subjects who received an injection volume ≤ 1.8 mL (17.4% and 8.7%, respectively). No serious AEs related to study treatment were reported.

## 4 Discussion

HA_SHA_ has previously been demonstrated to be safe and effective for chin augmentation and correction of chin retrusion in a pivotal clinal investigation [2]. However, real‐world patients often require treatments across multiple anatomical locations. To meet this need, this post‐market study evaluated the clinical performance of HA_SHA_ for chin augmentation alone (Group 1), in combination with HA_DEF_ for lower face (Group 2), and additionally with HA_LYF_ for midface (Group 3).

A consensus paper by experienced plastic surgeons and dermatologists previously outlined the safe and optimal use of HA_SHA_ in real world practice [3]. The recommended injection approaches, based on patient needs and treatment goals, were also considered in the design of the study presented here. The study therefore employed the Shayping Technique, which includes three treatment algorithms for diverse aesthetic needs, providing standardized guidance for clinical practice.

Results of the current study demonstrated a 100% GAIS responder rate in all groups at Weeks 4 and 8 (end of study) by both the investigator and subjects (Figure 2). These positive outcomes were further supported by high overall subject and Investigator satisfaction in all treatment groups (Figures 3 and 4) indicating that treatment with HA_SHA_ alone or in combination with HA_DEF_ and HA_LYF_ was effective in achieving the desired results, with minimal dissatisfaction. This suggests that the treatment results using the Shayping Technique were well received and successful in improving facial features and overall appearance.

The safety profile of all treatment combinations was also favorable, with mainly mild or moderate treatment‐related events and no serious AEs related to study product. The most common AEs, implant site pain and swelling (Table 5), were particularly common in subjects receiving higher injection volumes. The relationship between higher injection volumes and increased occurrence of AEs was also observed in the pivotal clinical investigation evaluating HA_SHA_ for augmentation and correction of chin retrusion [2]. This underscores the need for careful consideration of injection volumes in clinical practice. However, the current study reported no cases of implant site masses or nodules, potentially reflecting the relatively low median volume (1.8 mL) of HA_SHA_ used.

The findings from this study, particularly considering the high GAIS responder and satisfaction rates among both subjects and investigators, indicate that HA_SHA_, alone or in combination with HA_DEF_ and HA_LYF_, is effective in achieving desired aesthetic outcomes and can be confidently used in combination in clinical practice. The study also highlights the importance of considering injection volumes to minimize AEs, providing valuable guidance for practitioners to optimize treatment protocols and enhance patient safety, and supports the notion that combining different products can yield superior results in addressing diverse aesthetic needs. The absence of implant site masses or nodules, despite varying injection volumes, suggests that HA_SHA_ is well‐suited for achieving natural‐looking results without compromising safety. These findings can inform future research on injectable treatments and help refine theoretical models of facial aesthetics.

Despite the valuable insights gained, this post‐market study has some limitations. For example, the relatively small sample size may limit the generalizability of the findings and larger studies are needed to confirm these results across a broader population, and should aim to include a more diverse population (subjects of Asian, African and other origins). Furthermore, this was not a randomized controlled study and as such there may have been inherent bias in data collection and reporting, as real‐world settings can introduce variability that is not present in controlled clinical trials. The follow‐up period of 8 weeks may not capture long‐term outcomes and potential late‐onset AEs, therefore a follow‐up phase (e.g., 6–12 months) would be required to assess longevity of results and late‐onset AEs more prominently. Although the study highlights the impact of injection volumes on AEs, the variability in volumes used may introduce confounding factors that affect the interpretation of results.

In conclusion, depending on the subject's need, standalone treatment with HA_SHA_ for chin augmentation or in combination with HA_DEF_ and HA_LYF_ for a comprehensive lower face and midface lift was safe and effective, with 100% aesthetic improvement at 8 weeks post‐injection. The Shayping Technique successfully improves facial features and overall appearance and can be confidently used in clinical practice.

## Author Contributions

Andreas Nikolis, Jason K. Rivers, Nathan Rosen were clinical investigators in the study and contributed to patient recruitment and data collection. Felipe Weinberg, Inna Prygova, and Torun Bromée contributed to study design. All authors contributed to data interpretation, and reviewed and approved the manuscript prior to submission.

## Funding

This work was supported by Galderma.

## Ethics Statement

Prior to enrollment of any subjects, the clinical trial was approved by the appropriate institutional review boards on the following dates:

**Table:** 

WCIRB Research Ethics Board		
Executive Chair: Bridget Brave, JD		
Site	Principal Investigator	Research Ethics Board
		Approval Date
8754	Jason Rivers	07 May 2024
8690	Andreas Nikolis	03 May 2024
8379	Nathan Rosen	03 May 2024

All subjects in the study signed an informed consent form prior to participation. Subject approval for publication of photographs was included in the consent form to participate in the study.

## Conflicts of Interest

Andreas Nikolis is a paid consultant, speaker, and clinical trial investigator for Galderma, Allergan, Prollenium, and Merz. Jason K. Rivers is an advisory board member, speakers' bureau member, and investigator for AbbVie/Allergan; investigator for Galderma; advisory board member, speakers' bureau member, paid consultant, and investigator for Leo Pharma; investigator for Medytox; investigator for Pfizer; founder, stockholder of Riversol Skin Care Solutions Inc. Nathan Rosen is a clinical trial investigator for Galderma; investigator, consultant, speaker, and advisory board member for AbbVie; investigator for Revance; investigator, speaker, and advisory board member for Merz. Felipe Weinberg, Inna Prygova, and Torun Bromée are employees of Galderma.

## Data Availability

Research data are not shared.

## References

[1] Restylane Shaype IFU CANADA, accessed June 20, 2025, https://www.galderma.com/sites/default/files/2025‐03/90‐74745‐01_IFU_Restylane_Shaype‐2023.pdf.

[2] A. Nikolis , S. Humphrey , J. K. Rivers , et al., “Effectiveness and Safety of a New Hyaluronic Acid Injectable for Augmentation and Correction of Chin Retrusion,” Journal of Drugs in Dermatology 23, no. 4 (2024): 255–261, 10.36849/JDD.8145. 38564392

[3] A. Nikolis , K. Beleznay , V. Bertucci , et al., “Expert Recommendations on the Use of a New Hyaluronic Acid Injectable for the Aesthetic Treatment of the Chin and Lower Face,” Journal of Drugs in Dermatology 24, no. 1 (2025): 70–78, 10.36849/JDD.8593. 39761147

[4] Restylane Defyne IFU Canada, accessed June 20, 2025, https://www.galdermaaesthetics.com/ca/sites/default/files/2022‐04/Restylane%20Defyne.pdf.

[5] Restylane Lyft Lidocaine IFU Canada, accessed June 20, 2025, https://www.galdermaaesthetics.com/ca/sites/default/files/2022‐04/Restylane%20Lyft.pdf.

[6] Y. Xie , H. Zhao , W. Wu , et al., “Chin Augmentation and Treatment of Chin Retrusion With a Flexible Hyaluronic Acid Filler in Asian Subjects: A Randomized, Controlled, Evaluator‐Blinded Study,” Aesthetic Plastic Surgery 48, no. 5 (2024): 1030–1036, 10.1007/s00266-023-03812-2. 38315229 PMC10980616

[7] Y. Xie , W. Wu , J. Xu , et al., “A Randomized, Multicenter Study on a Flexible Hyaluronic Acid Filler in Treatment of Moderate to Severe Nasolabial Folds in a Chinese Population,” Journal of Cosmetic Dermatology 21, no. 10 (2022): 4288–4293, 10.1111/jocd.14914. 35279948

[8] K. Marcus , A. Moradi , J. Kaufman‐Janette , et al., “A Randomized Trial to Assess Effectiveness and Safety of a Hyaluronic Acid Filler for Chin Augmentation and Correction of Chin Retrusion,” Plastic and Reconstructive Surgery 150, no. 6 (2022): 1240e–1248e, 10.1097/PRS.0000000000009733. PMC9698183 36126213

[9] L. Baumann , R. A. Weiss , S. Grekin , et al., “Comparison of Hyaluronic Acid Gel With (HARDL) and Without Lidocaine (HAJUP) in the Treatment of Moderate‐To‐Severe Nasolabial Folds: A Randomized, Evaluator‐Blinded Study,” Dermatologic Surgery 44, no. 6 (2018): 833–840, 10.1097/DSS.0000000000001424. 29799827

[10] X. Wang , Y. Wu , B. Li , X. Mu , and L. Li , “Lifting the Midface Using a Hyaluronic Acid Filler With Lidocaine: A Randomized Multi‐Center Study in a Chinese Population,” Journal of Cosmetic Dermatology 21, no. 12 (2022): 6710–6716, 10.1111/jocd.15286. 35925834

[11] X. Wang , B. Li , and Q. Li , “Restylane Lyft for Aesthetic Shaping of the Nasal Dorsum and Radix ‐ A Randomized, No‐Treatment Control, Multi‐Center Study,” Plastic and Reconstructive Surgery 150, no. 6 (2022): 1225–1235, 10.1097/PRS.0000000000009732. 36103665 PMC9698109

